# Image-guided surgery and medical robotics in the cranial area

**DOI:** 10.2349/biij.3.1.e11

**Published:** 2007-01-01

**Authors:** G Widmann

**Affiliations:** Department of Radiology, Innsbruck Medical University, Anichstr, Austria

**Keywords:** image-guided surgery, mechatronic surgical tools, medical robotics, image-to-patient registration accuracy

## Abstract

Surgery in the cranial area includes complex anatomic situations with high-risk structures and high demands for functional and aesthetic results. Conventional surgery requires that the surgeon transfers complex anatomic and surgical planning information, using spatial sense and experience. The surgical procedure depends entirely on the manual skills of the operator. The development of image-guided surgery provides new revolutionary opportunities by integrating presurgical 3D imaging and intraoperative manipulation. Augmented reality, mechatronic surgical tools, and medical robotics may continue to progress in surgical instrumentation, and ultimately, surgical care. The aim of this article is to review and discuss state-of-the-art surgical navigation and medical robotics, image-to-patient registration, aspects of accuracy, and clinical applications for surgery in the cranial area.

## INTRODUCTION

Surgery in the cranial area includes operations of the fronto-zygomatico-maxillary complex, nasal cavity, paranasal sinuses, ear, and the skull base that have close proximity to highly critical structures such as nerves, vessels, the eye, cochlear and labyrinth organ, or the brain. Such operations often require re-establishing functional and aesthetic anatomy by repositioning displaced skeletal elements, or by grafting and contouring abnormal bony contours and transplants [1-5]. The need for accurate preoperative determination of the proposed surgical procedure is essential, and excellent intraoperative orientation and manual skills are required for surgical precision and reliable protection of vital anatomic structures [6-12]. Next generation surgical systems should explore and enhance imaging or manipulation, the two basic components of a surgical procedure [14]. The development of image-guided surgery provides new revolutionary opportunities by integration of presurgical 3D imaging, obtained by computed tomography (CT) or magnetic resonance imaging (MRI), and intraoperative manipulation through three fundamental issues [4,15,16]:

1. Localisation - determination of a target’s locus (for example, tumour, foreign body, and so on) that defines a task the surgeon performs,
2. Orientation - information on current location on the patient’s anatomy that defines where the surgeon (with respect to the surgical tool) is operating, and
3. Navigation - the process of (passive) guidance to reach a desired target from the current location (for example, biopsy, tumour resection, bone segment manipulation, implant positioning, and so on).

As a logical extension of image-guided surgery, the development of mechatronic surgical tools, tele-manipulated robotic arms, and semi- or fully- automated surgical robots are beginning to introduce the next revolution [17,18].

## SURGICAL NAVIGATION SYSTEMS

Surgical navigation systems generally consist of a (transportable) work station, a monitor, a graphical user interface with software to plan and guide therapy, and a position measuring system (a three-dimensional coordinate-detection or tracking system, which can be either mechanical, electromagnetic, or optical) [6,12,19-22]. By providing a spatial coordinate system relative to the patient's anatomy (see chapter on image-to-patient transformation), the actual position of a probe or tracked surgical tool is shown with respect to cross-sectional images of the preoperative dataset (see chapter on image guidance).

### Mechanical navigation systems

A mechanical navigation system consists of an articulated arm with six degrees of freedom [23-26]. Calculation of position is based on measurement of temperature changes recorded by a semiconductor temperature sensor within the gear of movable angles. As the spatial system is entirely self-referential, rigid fixation of both the patient and the navigation arm is an important prerequisite [19,20,23,24,27].

The advantages of the mechanical systems are acceptable precision, low susceptibility to failure, and sterile covering with a tube [21,23,25,29]. The disadvantages are impractical handling during some surgeries, restricted range (circa 60 cm), and mobility as well as the space requirements in the operating table [6,19,25,27]. Due to their bulkiness, the mechanical systems have been generally replaced by more flexible electromagnetic and optical navigation systems.

### Electromagnetic navigation systems

The position of electromagnetic navigation is measured by detecting of magnetic field changes with coils [19,21,29]. The electromagnetic transmitter is located near the operative site and the receiver is inside the surgical instrument. The advantages of electromagnetic navigation systems are the use of very small detector coils, absence of visual contact between instrument and sensor system, rapid computation of the signals, and easy sterilisation [21,25].

However, due to interference by external magnetic fields and metal objects, particularly those associated with drilling and sawing tools [12,21,30,31], incorrect position sensing of up to 4 mm may occur . To reduce the incorrect position sensing special titanium or ceramic instrument set is required [19,20,32]. Electromagnetic navigation systems are relatively contraindicated for use of patients with pacemakers and cochlear implants [29].

### Optical navigation systems

Optical based systems are used for intra-operative navigation [4,16,21,25,33,34]. Position calculation is provided by a minimum of three infrared diodes or passive light reflecting reference elements mounted to the registered patient using dynamic reference frame (DRF) and the surgical tool (tracker), and recognition of the obtained patterns with a stereotactic camera. The advantages of optical navigation systems are high technical accuracy in the range of 0.1-0.4 mm [35,36], convenient handling, and easy sterilisation. The disadvantages are the necessity of constant visual contact between camera-array, DRF and instruments, and the potential susceptibility to interference through light reflexes on metallic surfaces in the operating environment [21,25,37-39].

## MEDICAL ROBOTICS

Robots are generally defined as computer controlled devices with five to six degrees of freedom that can execute complex movements with high accuracy [14,40]. Medical robots can be classified based on technology, application, or role [14].

Using a technology-based classification, two groups of systems that differ substantially from each other can be distinguished:

- telemanipulators robots (not pre-programmed)
- pre-programmed surgical robots (automated or semi-automated)

Application-based taxonomy distinguishes robots on the basis of surgical disciplines and operative procedures. Role-based taxonomy distinguishes robots into three discrete categories:

- passive (the role of the robot is limited in scope or its involvement is largely low risk)
- restricted (the robot is responsible for more invasive tasks with higher risk but still restricted from essential portions of the procedure)
- active (the robot is intimately involved in the procedure and carries high responsibility and risk).

### Telemanipulated robots

Telemanipulated robots are non-autonomously working robotic arms (manipulator) that are controlled remotely by the surgeon using force-feedback joysticks or more advanced haptic devices (master console) [18,41]. Compared to conventional endoscopic arms with limited mechanical control, telemanipulated robots provide a greater degree of freedom and have a computer controlled men-machine interface that allows for automatically processing of the input for the manipulator system without active interaction by the surgeon for motion scaling, tremor filtering, indexing, and so on. [18,41-44].

### Pre-programmable surgical robots

Pre-programmable surgical robots can automatically or semi-automatically execute surgical tasks directly on the patient. These systems include:

- floor or operating table mounted robots with six degrees of freedom
- roof mounted modified surgical microscopes with generally six to seven active and one passive degree of freedom [45-49]

The surgeon in the operating theatre supervises the execution of the plan by the robot [7,50].

Interactive assistant robots are navigated tool support systems that carry, guide, and move surgical instruments. The robot is primarily moved passively by the surgeon but the robot can limit the degrees of freedom of the movements. Favourable positions can be saved and reached again with high precision. The surgeon has a spatial interval in which free movements are allowed, preventing movement into high-risk areas [21,40].

### Mechatronic surgical tools

As a separate development in surgical instrumentation, mechatronic surgical tools are dedicated to special tasks such as drilling or bone shaving [5,51,52]. These tools may include force feedback sensors to prevent bone perforation or navigated controlled systems that only work within a certain surgical accuracy threshold.

## IMAGE-TO-PATIENT TRANSFORMATION

Image-to-patient (IP) transformation or registration is the essential determination of a one-to-one mapping between the coordinates in the image data and those in the patient [53,54]. The registration procedure is based on anatomical landmarks (bone or skin), artificial markers (fiducials, bone affixed or skin applied), teeth supported registration templates, external registration frames, and laser surface scanning [12,25,55-60].

### Anatomical landmarks

Registration with anatomical landmarks uses clearly defined external (such as nasion, spina nasalis, tragi, medial canthi, mastoid, umbo, and so on) and/or internal landmarks [61,62]. However, precise identification of the landmarks in both the patient and the image dataset is subjective and depends on the experience of the operator [63]. Surface matching, which is done by touching about 40-80 points on the patient’s skin or bone, can refine anatomical registration [62,64]. However, this method is generally inaccurate and time-consuming.

### Fiducial markers

The advantage of fiducial markers over anatomical landmarks is the enhanced localisation accuracy on the image data and the patient. Consequently, registration with skin-applied fiducials is more accurate than registration with surface anatomical landmarks [65-67]. However, the use of skin-applied fiducials is associated with high logistics because the markers must be placed prior to data set acquisition and must be kept in their position until the patient enters the operating room. The time lag between imaging and surgery, and the sensitivity to skin shift can lead to unfavourable inaccuracies [25,56,63,68-70]. Bone-implanted fiducials provide invariant spatial registration points with the highest possible accuracy and generally serve as the reference gold standard in registration [21,53,66,68,71-73]. The drawbacks of bone-implanted fiducials are their invasiveness, the need for additional surgery, and possible major patient discomfort for which they should not be left in place for an extended period [55,63,70,71].

### Registration templates

Registration templates are non-invasive, denture fixed acrylic splints with integrated fiducial markers [36,39,60,71,74-81]. Proven accuracy similar to bone implanted fiducials is available for the regions of the maxilla, mandible, orbit and face [36,72,81]. Registration templates cannot be applied to edentulous patient, except when the templates are invasively secured to the underlying bone.

### Vogele-Bale-Hohner (VBH) mouthpiece / external registration frame

The Vogele-Bale-Hohner (VBH) vacuum mouthpiece is an individualised mouthpiece that can be objectively and rigidly secured against the maxilla with submillimetric repositioning control, that is regulated by the amount of negative pressure on the scale of a vacuum pump [56,82-84]. Alternatively, the VBH mouthpiece can be glued to an acrylic template, similar to registration templates. Compared to registration templates, where the markers are integrated in the template, an external registration frame is connected to the VBH mouthpiece. The VBH mouthpiece can be removed after registration [55,59,82,83,85-89]. The external registration frame allows for broad marker distribution around the entire head volume. Supported with exchangeable markers for CT/MRI/PET/SPECT, the external registration frame can serve as a single reference device for multimodal surgical navigation and fusion imaging [56,84,90-92].

### Laser surface registration

Laser surface registration is based on projection of visible laser beams on the patient’s skin [67-70,93]. The skin reflections are detected by a camera array and a virtual three-dimensional matrix of the skin anatomy of the patient is generated. The matrix, which is an advanced surface-matching algorithm, is then matched to the surface matrix of the pre-operative image-data set.

Currently, up to 300,000 skin surface points can be registered. This allows the registration accuracy reach comparable values to bone markers or registration templates [67]. However, the shift of the patient’s skin surface or different tension in muscles of expression when performing CT-data acquisition and during preoperative and intraoperative recording, may lead to an invalid data set correlation [68,69,93]. Though the patient might to be continuously tracked during surgery, the original geometry of the facial soft tissue may be destroyed by intraoperative swelling, surgical cuts, or during repositioning osteotomies [21,33,69,94]. To compensate, a combination with dynamic reference frames must be available for intraoperative tracking after the initial laser registration has been reported [94]. Laser surface registration is unsuitable for surgery in the mandible but is expected to serve as a sufficiently stable and relatively invariable reference base for many applications in cranio-maxillofacial surgery [66,67,70, 93,94].

## IMAGE-GUIDANCE

For image-guidance, the correlation between the space coordinates of the image-data in the navigation system and the patient’s coordinates defined during registration are preserved during the surgical procedure. The coordinates are obtained by rigid fixation of the patient on the operating table, for example invasively via the Mayfield head clamp, or non-invasively via the vacuum mouthpiece based VBH head holder [56,64,95]. Alternatively, bone (invasive) or registration template (non-invasive) affixed DRFs are used for continuous patient tracking after initial registration [33,36,60,74,76].

During surgery, the navigation software indicates the actual real-time position of the tracked surgical tool within the patient’s presurgical 3D-data for intraoperative orientation, and shows the calculated accuracy of the tool’s position and angulations related to the predefined surgical plan. Integrated mechatronic surgical tools provide automatic on/off-regulation depending on the current position of the patient in relation to the planned working space or the connection of the drill speed to the operator accuracy. Integrated mechatronic surgical tools are immediate stopped when possible damage to vital structures occurs (= navigated control) [5,51,52]. In addition, the development of adjustable rigid aiming devices enables a steady linear approach to defined targets [56,85,96-98].

Visualisation of the navigation process is generally provided via the computer screen of the navigation system’s transportable workstation. A disadvantage of such a display is that the surgeon has to look up at the screen and therefore, cannot simultaneously view the surgical field [4,13,39,51,71].

In contrast, augmented reality (AR) provides navigational support by direct projection of segmented structures from the preoperative image data (surgical targets, resection lines, and planned implant position) to the patient. Therefore allowing complete interaction with the real world, while simultaneously making the virtual environment accessible [30,58,71,99,100-104,111]. AR can be based on monocular projection in the operating microscope or the binocular optics of a tracked surgical microscope projection for the purpose of building semitranslucent screens placed between the operating screen and the surgeon or the head mounted displays [4,22,30,58,71,99,100,105-110]. Recently, a promising AR concept using laser registration and stereotactic optical projection of tumour margins and osteotomy lines directly on the patient was presented. This concept does not necessitate navigation instruments [104,111,112].

## ASPECTS OF ACCURACY

### Terminology

Accuracy is of utmost importance for clinical application of image-guided surgery and medical robotics. Use of standardised terminology and measurement types is essential for correct understanding and comparability of accuracy reports [113].

Accuracy is qualitatively determined as the amount of approximation of the mean of the measurements to the true value (which refers to the term trueness) and quantitatively determined through the margin of error and the uncertainty of measurement, which is characterised by the variation of the mean value from several single measurements.

Precision is the inner accuracy of measurements obtained by repeated measurements (under the same circumstances and with the same measurement technique and system) and refers to the quantitative characterisation of the concision of the measuring instrument and its readout. Although often used as a synonym for accuracy, precision must be clearly distinguished from the term accuracy.

For evaluation of image-guided surgery, the suggested measurement types are as follows: [66,72,113-115]:

- Fiducial Localising Error (FLE): the error in locating the fiducial points.
- Fiducial Registration Error (FRE) : the error between corresponding fiducial points after registration
- Target Registration Error (TRE) : the error between corresponding points other than the fiducial points after registration
- Target Positioning Error (TPE): the error between the real position of the navigated surgical tool and the calculated position during the actual surgical procedure (TRE plus additional factors).

The best indicator for a navigation system’s or medical robot’s accuracy is represented by the TPE, but the definitive overall accuracy of the surgical procedure has to be ultimately evaluated by directly comparing the achieved surgical result to the initial planning data.

### Influential factors of accuracy

The overall accuracy of image-guided / robotic surgery depends on all systematic and non-systematic (random) errors, from the data-set acquisition to the surgical procedure [116]. The accurate linking of the virtual planning to the surgical site depends on the accuracy of the registration procedure, which includes limitations in the image space and the device space (see chapter image-to-patient transformation). Image quality depends on the image resolution as represented by the voxel size and slice thickness. The thinner the slice thickness and the smaller the voxel size, the higher is the accuracy of determining the centre of the fiducial markers (fiducial-based registration) or the accuracy of the calculated 3D surface model (surface based registration) [88,119,120]. In principle, multi detector CT is more accurate than MRI, because MRI is prone to inhomogeneities of the magnetic field and, due to the longer examination time, more susceptible to motion artefacts [64,117-119]. The arrangement of fiducial markers is a critical factor and it is important to use as many points as possible (although the return diminishes rapidly after five or six markers are used), avoid near-collinear configurations, and ensure that the centroid of the fiducial points is as near as possible to the target [12,54]. The typical feedback provided by the registration software is a measure of the degree of alignment of the points used in the registration. Unfortunately these measures show no direct correlation to the TRE and to reliably control the registration accuracy intraoperatively, the real error between the image and the patient’s anatomy has to be checked prior to surgery by a few independent markers not used for initial registration and/or by anatomic landmarks [10,12,36,39,77,93]. This can be performed with the probe of the navigation system by comparing the probe’s real position (device space) to the virtual position displayed on the computer screen (image space). The accuracy of the surgical transfer is dependant on the technical accuracy of the navigation system, mechatronic, semi-active, or active robotic system and the surgical application accuracy. Notably, human error is attributed to imaging, registration, and transfer errors, for which every step has to be carefully managed.

## CLINICAL APPLICATIONS

### Image-guided surgery

Successful clinical applications of image-guided surgery in the cranial area have been already described for many procedures, such as the following (neurosurgical procedures excluded): oral implant surgery [10,16,37,38,52,73,77,79,103,121], removal of tumours and foreign bodies [16,33,58,76,81,122], bone segment navigation [60,122,123], temporo-mandibular-joint surgery [74,124], biopsy [16], frameless stereotactic interstitial brachytherapy [28,87], percutaneous radio frequency ablation of the Gasserion ganglion in medically untreatable trigeminal neuralgia [88,95,125], functional endoscopic sinus surgery and skull base surgery [5,9,12,22,107,126-128]. Use of mechatronic surgical tools has been tested for navigate-controlled drilling in oral implant surgery [52] and shaving in functional endoscopic sinus surgery [13,51].

### Medical robotics

In the cranial area, robotic systems were considered to help the surgeon interactively with the following tasks [1,7,21,40,45,129]: (1) the drilling of holes with an automatic stop after penetrating the bone to protect the tissue lying deep to the bone, (2) the defined drilling of the implant bed for positioning of implants or bone fixtures for anaplastology, (3) the milling of the bone surfaces in plastic surgery according to a 3D-operation plan, (4) performing deep saw-cuts for osteotomies and allowing for the precise three-dimensional transportation of the subsequent bone segments or CAD/CAM (computer aided design / computer aided manufacturing) transplant, (5) the preoperative automatic selection of the necessary osteosynthesis plates, their bending by a special machine and their intraoperative positioning in defined positions, or (6) the automated guidance for non-flexible catheter implantation at brachytherapy.

Pre-clinical and clinical studies have been started around the millennium in Germany, France, USA and Japan for robot-assisted placement of craniofacial implants in ear anaplastology [130], resection of frontotemporal bone segments [131], implant fabrication combined with CAD/CAM technology in reconstructive surgery [21,79,131], model surgery in orthognatic surgery [26], passive guidance for the positioning of oral implants [133-135], and videoendoscopic ENT and skull base surgery [18,47-49,132].

### Cost-benefit ratio

Image-guided surgery is considered to be more accurate than standard surgery. Comparative studies in oral implant surgery indicate significantly more accuracy compared to the manual freehand procedure even if performed by experienced surgeons [79,136,137]. In addition, no significant difference between experienced surgeons and trainees was found, which demonstrates that image-guidance is a valuable means for achieving a predictable and reproducible result without heavy reliance on the clinician’s surgical experience [10,79,136,138]. In other procedures, such as percutaneous interventions (which are generally a “blind” surgical procedure), removal of foreign bodies, access to deep seated locations, orientation in complex and changed anatomic regions, etc., clear benefit of image-guidance is evident [4,12,16,33,128,143]. Generally, shorter operation time, safer manipulation around delicate structures and higher intraoperative accuracy have been reported [9,16,20,60,128,139,140]. Further, image-guidance may allow for more thorough surgical resection and potentially decreasing the need for revision procedures [140].

In a large clinical study for image-guided ENT surgery, it was found that image-guidance can provide additional relevant information that was not available to the surgeon solely by virtue of his existing knowledge and that every second application of the navigation system may lead to a change in surgical strategy [5]. Accordingly more benefit is obtained from additional orientation and resulting cognitive relief at the moment of stressed and distracted surgical situations. Another clinical study including 158 surgical procedures in cranio-maxillo-facial surgery showed high to very high medical benefits for image-guided biopsies, punctures of the trigeminal ganglion, removal of foreign bodies, osteotomies of the facial skeleton, arthroscopies of the temporomandibular joint and positioning of dental implants [16].

Image-guided surgery is more expensive than the standard procedure (navigation systems cost about USD 60,000 to USD 200,000) and requires presurgical imaging with registration elements, intraoperative image-to-patient registration and specialised equipment for tool tracking. However, these systems can be used for a wide range surgical procedures in different medical specialities [56,57,59,83-85,89,144] and thus may represent a valuable acquisition for an institution [16,33]. A further beneficial aspect is the associated automatic and complete electronic documentation of the intervention [16,116].

Robots are expected to be more accurate and more reliable than a human being. Robots can work as part of an interactive system, are immune to radiation and can be automatically programmed for documentation, evaluation and training protocols [14,40,45,46,129]. Except for very few cases, surgical robots will not execute operations fully autonomously but will support the physician to achieve optimal results [1,7,21,40,44, 45,129,141].

Considering the advantages mentioned above, image-guided surgery and medical robotics may have a positive cost/effort–benefit ratio, depending on the individual surgical task and the developmental stage of each system. The necessity of special knowledge for this technology is indisputable and the relationship between cost and benefit may additionally be dependent on familiarity and availability [15,113].

## CONCLUSION

Due to the complex anatomic situations with high-risk structures and the high demands for functional and aesthetic results, surgery in the cranial area is a prototype for application of image-guided surgery and medical robotics. Successful clinical use has been already described for many different procedures and clear benefit is proved in terms of intraoperative orientation, surgical accuracy, safety and reduced operation time. The development of mechatronic surgical tools may additionally improve safety and surgical accuracy. For appropriate clinical application of image-guided surgery, it is important that the surgeon is aware of all influential factors of accuracy and the maximum error of each system / technique regarding the required surgical accuracy for the individual operation.

In the future, surgical navigation with integration of intraoperative imaging, improved augmented reality techniques, sophisticated mechatronic surgical tools and new robotic developments which are smaller, less expensive and easier to operate will enable continued progress in surgical instrumentation, and ultimately, surgical care.
